# Identification of several plasma proteins whose levels in colorectal cancer patients differ depending on outcome

**DOI:** 10.1096/fba.2019-00062

**Published:** 2019-11-23

**Authors:** Matilda Holm, Sakari Joenväärä, Mayank Saraswat, Harri Mustonen, Tiialotta Tohmola, Ari Ristimäki, Risto Renkonen, Caj Haglund

**Affiliations:** ^1^ Department of Surgery Faculty of Medicine University of Helsinki and Helsinki University Hospital Helsinki Finland; ^2^ Department of Pathology Faculty of Medicine University of Helsinki and Helsinki University Hospital Helsinki Finland; ^3^ Translational Cancer Medicine Research Program Faculty of Medicine University of Helsinki Helsinki Finland; ^4^ Applied Tumor Genomics Research Program Faculty of Medicine University of Helsinki Helsinki Finland; ^5^ Transplantation Laboratory Haartman Institute University of Helsinki Helsinki Finland; ^6^ HUSLAB Helsinki University Hospital Helsinki Finland; ^7^ Department of Laboratory Medicine and Pathology Mayo Clinic Rochester MN USA; ^8^ Department of Biosciences Faculty of Biological and Environmental Sciences University of Helsinki Helsinki Finland

**Keywords:** mass spectrometry, outcome, plasma proteome, proteomics, stage

## Abstract

Colorectal cancer (CRC) stands for 10% of the worldwide cancer burden and has recently become the second most common cause of cancer death. The 5‐year survival rate depends mainly on stage at diagnosis. Mass spectrometric proteomic analysis is widely used to study the plasma proteome, which is complex and contains multitudes of proteins. In this study, we have used Ultra Performance Liquid Chromatography‐Ultra Definition Mass Spectrometry (UPLC‐UDMS^E^)‐based proteomics to analyze plasma samples from 76 CRC patients. We identified several plasma proteins, such as CP, TVP23C, FETUB, and IGFBP3, of which altered levels led to significant differences in survival, as seen by Cox regression and Kaplan‐Meier analysis. Additionally, during Cox regression analysis, samples were adjusted for age and/or tumor stage, enabling stringent analysis. These proteins, although in need of further validation, could be of use during patient follow‐up, as their levels can non‐invasively be measured from blood samples, and could be of use in predicting patient outcome. Several of these proteins additionally have roles in metabolism and inflammation, two processes central to the development and progression of cancer, further indicating their importance in cancer.

**Abbreviations**
C3: complement C3
CFH: complement factor H
CNK3/IPCEF1: CNK3/IPCEF1 fusion protein
CP: ceruloplasmin
CRC: colorectal cancer
DEPDC1: DEP domain-containing protein 1A
EPHA5: ephrin type-A receptor 5
FDR: false discovery rate
FETUB: fetuin-B
HR: hazard ratio
GPLD1: phosphatidylinositol-glycan-specific phospholipase D
IGFBP3: insulin-like growth factor-binding protein 3
LCAT: phosphatidylcholine-sterol acyltransferase
MBL2: mannose-binding protein C
MORC2: MORC family CW-type zinc finger protein 2
PIWIL4: piwi-like protein 4
SIPA1L1: signal-induced proliferation-associated 1-like protein 1
TVP23C: golgi apparatus membrane protein TVP23 homolog
UPLC-UDMSE: Ultra Performance Liquid Chromatography-Ultra Definition Mass Spectrometry

## 1 INTRODUCTION

In 2018, over 1.8 million new cases of colorectal cancer (CRC) and closer to 900 000 deaths were estimated to have occurred, meaning that CRC stands for 10% of the global cancer burden. CRC is the third most common cancer and has risen to become the second most common cause of cancer death, behind only lung cancer. The highest incidence rates are found in Europe, North America, and Oceania, with incidence rates being lower in Africa, South‐Central Asia, and Central America. The overall 5‐year survival rate for CRC patients is around 65%, and the 5‐year survival rate depends heavily on stage at diagnosis.^1^, ^2^ Per stage, 5‐year survival is over 90% for stage I disease, 82.5% for stage II disease, 59.5% for stage III disease, and only 8.1% for stage IV disease. Around 20% of patients with stage II local disease and no lymph node metastasis will develop recurrence, and of those with lymph node metastasis but no distant metastasis, around half will develop recurrence.^3^, ^4^

Stage at diagnosis is the most important prognostic factor for CRC.^5^ The anatomically based TNM staging system, although widely used, struggles to clearly distinguish groups of patients with different prognosis among stage II and III CRC patients, particularly in those who receive adjuvant chemotherapy.^6^ For stage III CRC patients, an overall survival benefit has been established for fluorouracil‐based chemotherapy, although for patients with stage II CRC, the use of adjuvant chemotherapy is controversial. Studies have failed to demonstrate a significant overall survival benefit in stage II CRC patients who receive adjuvant therapy, and current guidelines do not support its routine use.^7^, ^8^

Currently, mass spectrometric proteomic analysis is widely used in efforts to analyze samples such as plasma. The plasma proteome is dynamic and reflects the state of the host due to the perfusion of organs. It therefore also reflects the presence of diseases such as cancer which can add, subtract, or modify circulating proteins.^9^, ^10^ Proteomic analysis by mass spectrometry therefore enables the identification of differentially expressed proteins in plasma.^11^, ^12^ The plasma proteome is also ideal to study due to its complexity, as it encompasses many different types of proteins. True plasma proteins that carry out their functions in the circulation, proteins secreted by tissues and tumors, proteins that serve as messengers between tissues, temporary passengers, proteins that leak from tissues as a result of cell death or damage, and foreign proteins are all part of the plasma proteome.^13^

Previous proteomic studies of CRC have mainly investigated serum or plasma proteins that could be of use in the early detection and diagnosis of CRC by comparing samples from healthy controls to samples from CRC patients.^14^, ^15^, ^16^, ^17^ Several studies have also identified proteins of value as potential prognostic markers for CRC patients by studying tissue samples.^18^, ^19^ One study by Surinova et al employed proteomic profiling of 80 glycoprotein biomarker candidates and subsequently discovered a six‐protein biomarker signature that could predict patient survival.^20^ In this study, we have used Ultra Performance Liquid Chromatography‐Ultra Definition Mass Spectrometry (UPLC‐UDMS^E^)‐based proteomics to analyze plasma samples from 76 CRC patients. The aim of this study was to discover proteins whose levels differed significantly between patients with good and poor long‐term survival, as these proteins may be of future clinical utility.

## 2 MATERIALS AND METHODS

### 2.1 Patient samples

This study used preoperative plasma samples from a total of 76 CRC patients who underwent surgical resection with curative intent at the Department of Surgery, Helsinki University Hospital, between 2000 and 2007. Plasma samples were stored at −80°C until processed as described below. Patients with a previous history of non‐colorectal cancer, hereditary nonpolyposis colorectal cancer, familial adenomatous polyposis, ulcerative colitis, Crohn's disease, or mucinous tumors were deliberately excluded from this study. Detailed patient characteristics are given in Table S1. The clinical data were obtained from patient records, the survival data from the Population Register Centre of Finland, and the cause of death for all the deceased from Statistics Finland. Written informed consent was obtained from all patients prior to collecting samples. This study was approved by the Surgical Ethics Committee of Helsinki University Hospital (Dnro HUS 226/E6/06, extension TMK02 §66 17.4.2013) and carried out in accordance with the Declaration of Helsinki.

### 2.2 Sample processing and digestion

The plasma samples were processed as previously described^21^ and as follows. Samples were thawed and top 12 protein depletion was performed using the TOP12 protein depletion kit (Pierce, ThermoFisher) according to the manufacturer's instructions. The total protein concentration was determined using the Pierce BCA assay kit (Pierce, ThermoFisher). The amount of plasma equivalent to 100 µg of protein was aliquoted and dried using a SpeedVac (Savant, ThermoFisher), and the dried plasma was then dissolved in 35 µL Tris buffer (50 mmol/L, pH 7.8) containing 6 mol/L urea. 1.8 µL of dithiothreitol (DTT, 200 mmol/L) was then added to each sample and the samples were shaken for 1 hour at room temperature, after which 7 µL of iodoacetamide (200 mmol/L) was added to each sample. Samples were again shaken for 1 hour at room temperature, after which 7 µL of DTT (200 mmol/L) was added to each sample to quench excess iodoacetamide and prevent overalkylation. The samples were shaken for 1 hour at room temperature, after which they were diluted by adding 270 µL mQ water per sample. Trypsin was added at a ratio of 1:50 trypsin to protein and the samples were digested at 37°C overnight. The next day, 30 µg of tryptic peptides were cleaned using C18 spin columns (Pierce, ThermoFisher) and the cleaned peptides were dissolved in 86 µL of 0.1% formic acid containing 12.5 fmol/µL of Hi3 spike‐in standard peptides (Waters) for quantification.

### 2.3 Ultra performance liquid chromatography‐ultra definition mass spectrometry and quantification

#### 2.3.1 UPLC‐UDMS^E^

UPLC‐UDMS^E^ was performed as previously described.^21^ Data were acquired in data‐independent acquisition fashion using UDMSE mode using a Synapt G2‐S HDMS (Waters Corporation) and collected in the range of 100‐2000 *m/z*, scan time one second, IMS wave velocity 650 m/s. Collision energy was ramped from 20 to 60 V and calibration was performed with sodium iodide clusters over a mass range of 50‐2500 *m/z* by infusing 2 µg/µL sodium iodide solution in 50/50 2‐propanol/water into the mass spectrometer. 10% of the samples were run in triplicate and the median coefficient of variation (%CV) of the dataset was 4.36%.

### 2.4 Data analysis

Data analysis and label‐free quantification were performed as previously described.^21^, ^22^, ^23^ In summary, the raw files were imported to Progenesis QI for proteomics (Nonlinear Dynamics). Post‐acquisition mass correction was done when the raw data were imported into Progenesis with a lock mass ion of M+H+ 556.2771 *m/z*. Leucine enkephalin (C25H37O7, 1 ng/µL in 50:50 acetonitrile:water + 0.1% formic acid) was infused into the reference sprayer at 300 nL/min for this purpose. Default parameters were used for peak picking and alignment, while the peptide identification was done against Uniprot human FASTA sequences (release 2018_04). A ClpB protein sequence (CLPB_ECOLI (P63285)) was inserted for label‐free quantification. Fixed modification at cysteine (carbamidomethyl) and variable at methionine (oxidation) were used. Trypsin was used as a digesting agent, with one missed cleavage allowed. Fragment and peptide error tolerances were set to automatic settings, and the false discovery rate (FDR) was set to less than 2%. The default parameters for ion fragments required to identify peptides were used.

The parsimony principle was used to group the proteins and peptides unique to the protein were also reported. Progenesis QI for proteomics does not follow a strict parsimonious approach due to over‐stringency, which has been previously noted.^24^ In the case of a conflict where two proteins were found with common peptides, the protein with fewer peptides is absorbed into the protein with more peptides. All relevant proteins are listed as a group under the lead protein with the highest coverage or score if the coverages of two or more proteins are equal. Quantitation was performed using the lead identity peptide data. Further details can be found on the Nonlinear Dynamics’ website (://www.nonlinear.com).

### 2.5 Further analysis

The mass spectrometry proteomics data have been deposited to the ProteomeXchange Consortium via the PRIDE^25^, ^26^ partner repository with the dataset identifier PXD013150 and 10.6019/PXD013150. Survival was analyzed using the Kaplan‐Meier method, log‐rank tests, and Cox proportional hazards regression analysis. Cox proportional hazards regression analysis was used for age‐ and stage‐adjusted analyses. Cut‐off values were determined by maximizing the absolute value of Youden's index from receiver operating characteristics curves, thereby assigning equal weight on sensitivity and specificity.

## 3 RESULTS

### 3.1 Protein identification and analysis

In this study, we analyzed plasma samples from 76 CRC patients (after the exclusion of one sample, marked in Table S1, that failed to digest properly). We quantified 224 proteins that contained two or more unique peptides, and these proteins were subsequently used for further analysis. All 224 proteins with their relevant data are given in Table S2.

### 3.2 Analysis of all samples

Univariate Cox regression analysis was performed on all 224 proteins quantified (Table S3). Five proteins passed the cut‐off of a Cox regression *P*‐value of <.05: cDNA FLJ55673, highly similar to Complement factor B, ceruloplasmin (CP), golgi apparatus membrane protein TVP23 homolog (TVP23C), fetuin‐B (FETUB), and insulin‐like growth factor‐binding protein 3 (IGFBP3). These proteins are given with their relevant details in Table 1. The hazard ratio (HR) for these proteins was <1, indicating that high plasma levels are correlated with favorable prognosis. Additionally, all samples were also adjusted for age and stage and analyzed by Cox regression. The top five proteins according to their Cox regression *P*‐value are given in Table 2. Only one protein, FETUB, had a *P*‐value of <0.05 (Table 2).

**Table 1:** The proteins with significant *P*‐values when all samples were analyzed by univariate Cox regression

Accession	Protein name	Gene name	*P*‐value	Hazard ratio (HR)	95% CI (lower)	95% CI (upper)
B4E1Z4;P00751	cDNA FLJ55673, highly similar to Complement factor B		0.00	0.59	0.421	0.840
P00450;Q96CS3;Q96L14;Q99551;Q9BRC7	Ceruloplasmin	CP	0.03	0.70	0.505	0.968
Q96ET8;Q9NYZ1	Golgi apparatus membrane protein TVP23 homolog	TVP23C	0.03	0.71	0.524	0.970
Q9UGM5	Fetuin‐B	FETUB	0.04	0.69	0.488	0.984
P17936	Insulin‐like growth factor‐binding protein 3	IGFBP3	0.05	0.73	0.533	0.993

Note

Accession, protein and gene name, *P*‐value, HR, and lower and upper 95% CI are given in the table.

**Table 2:** The top five proteins according to p‐value when all samples were analyzed by univariate Cox regression and adjusted for age and stage

Accession	Protein name	Gene name	*P*‐value	HR	95% CI (lower)	95% CI (upper)
Q9UGM5	Fetuin‐B	FETUB	0.033	0.67	0.462	0.968
P04180	Phosphatidylcholine‐sterol acyltransferase	LCAT	0.053	0.72	0.516	1.004
B4E1Z4;P00751	cDNA FLJ55673, highly similar to Complement factor B		0.065	0.70	0.474	1.022
P04196	Histidine‐rich glycoprotein	HRG	0.075	0.74	0.530	1.031
Q92777	Synapsin‐2	SYN2	0.077	0.78	0.585	1.028

Note

One protein, fetuin‐B, had a *P*‐value of less than 0.05.

**Figure 1:**
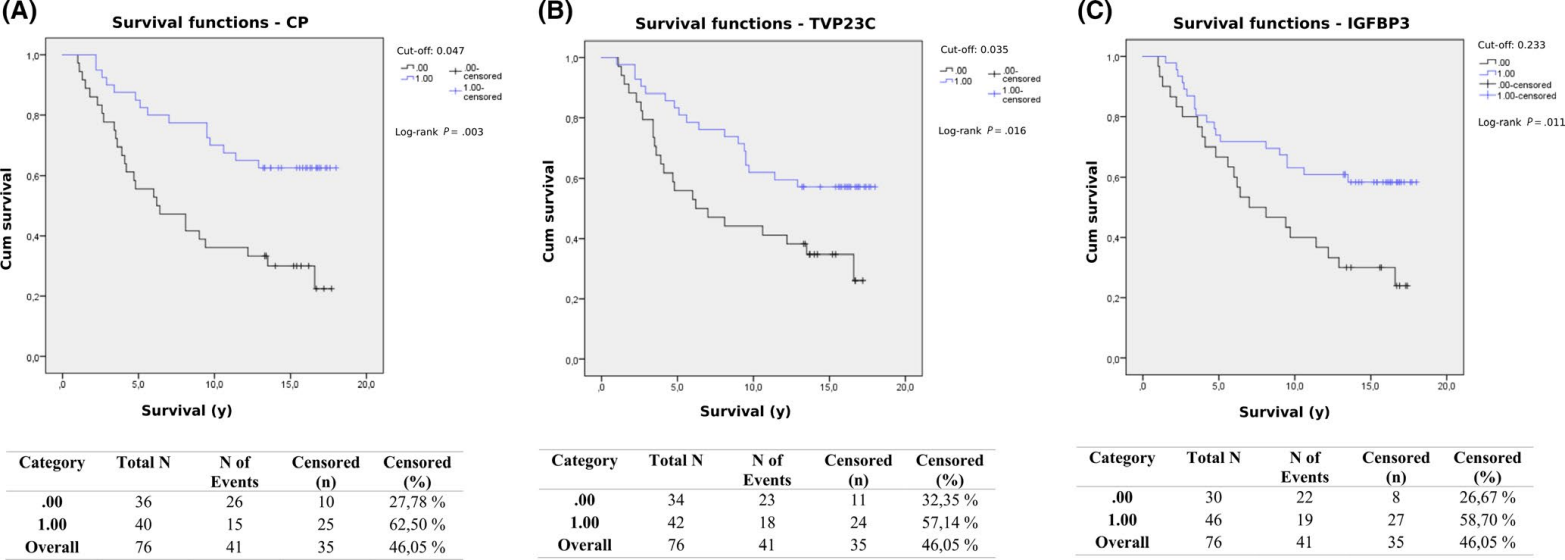
The Kaplan‐Meier curves for CP (A), TVP23C (B), and IGFBP3 (C) when all samples were analyzed. The cut‐off was set at the maximum of Youden's index for each protein. In all figures, the long‐term survival of CRC patients with plasma levels above the cut‐off was better than for patients with levels below

Kaplan‐Meier curves were generated for each of the five proteins, including the cDNA identified (Figure S1A), with the cut‐off set at the maximum of Youden's index for each protein. The differences in survival depending on plasma levels of these proteins were significant (*P* < .05) as analyzed by log‐rank tests for all proteins except FETUB (*P* = .068) (Figure S1B). The Kaplan‐Meier curve for CP is shown in Figure 1A, for TVP23C in Figure 1B, and the curve for IGFBP3 is shown in Figure 1C. In all figures, the long‐term survival of patients with plasma levels above the cut‐off was better than for patients with levels below. The differences in outcome between the groups became even clearer as time passed.

### 3.3 Analysis of stage II samples

Cox regression analyses were also performed when samples were divided according to tumor stage (II or III). Eight proteins, given in Table S4, had significant p‐values when only stage II samples were analyzed by Cox regression. Higher plasma levels of the same cDNA identified when all samples were compared, as well as CP and TVP23C, also predicted a better survival for stage II patients. Higher levels of two complement‐related proteins, complement factor H (CFH) and Complement C3 (C3), were also found to be linked to a favorable outcome. These samples were also adjusted for age and analyzed by Cox regression again, after which 11 significantly different proteins were identified (Table S5). Several of the proteins such as CP, TVP23C, CFH, and C3 had significant *P*‐values even without age‐adjustment, although other proteins such as piwi‐like protein 4 (PIWIL4) and DEP domain‐containing protein 1A (DEPDC1) had non‐significant p‐values prior to age‐adjustment.

**Figure 2:**
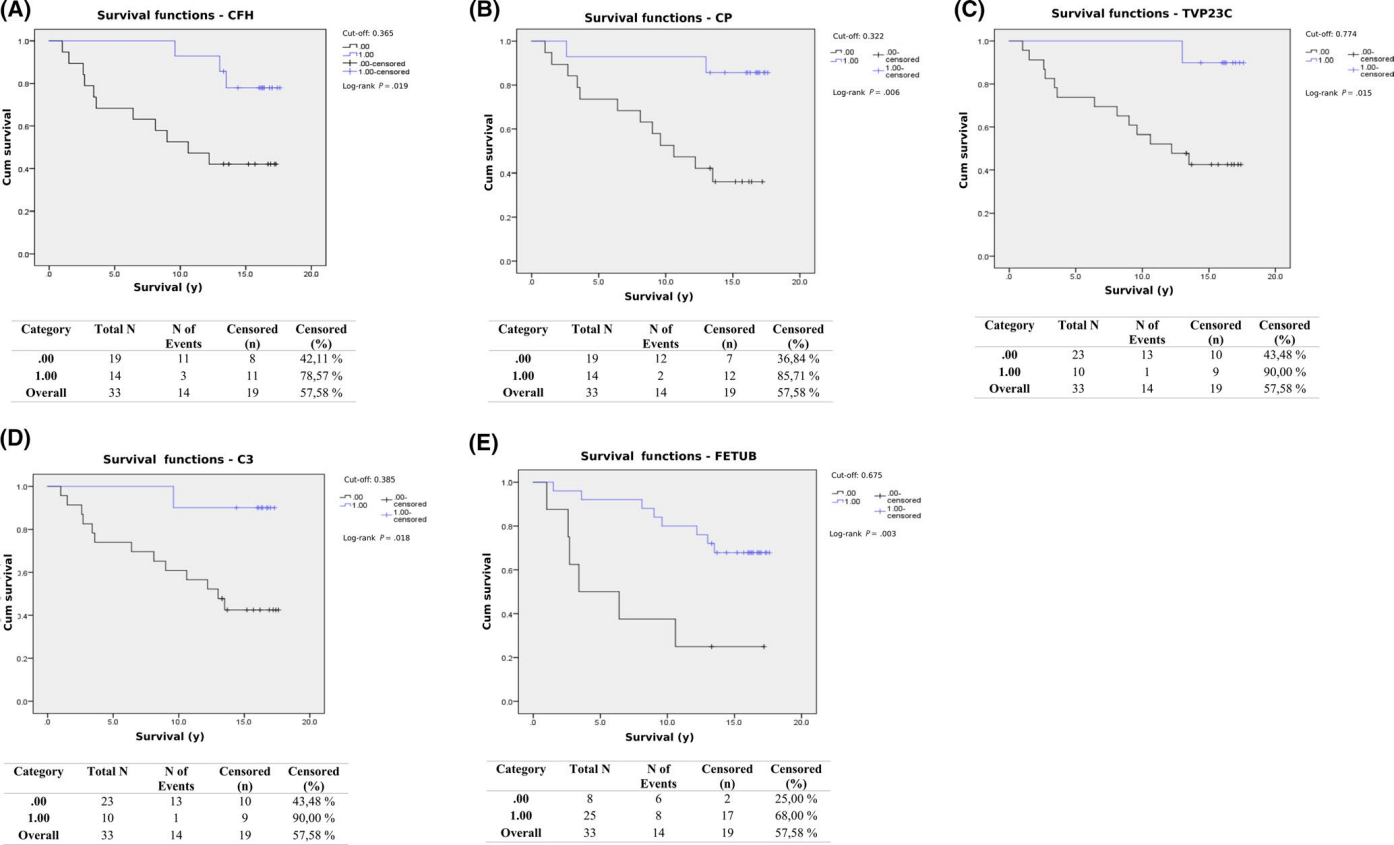
The Kaplan‐Meier curves for CFH (A), CP (B), TVP23C (C), C3 (D), and FETUB (E) when only stage II samples were analyzed. The cut‐off was set at the maximum of Youden's index for each protein. In all figures, the long‐term survival of stage II CRC patients with plasma levels above the cut‐off was better than for patients with levels below

Kaplan‐Meier curves were drawn with the cut‐off set at the maximum of Youden's index for each protein. The Kaplan‐Meier curve for CFH, shown in Figure 2A, shows that higher plasma levels of CFH are linked to better long‐term survival (*P* = .019). For CP (Figure 2B), TVP23C (Figure 2C), and C3 (Figure 2D), the differences in survival were also significant as seen in the Kaplan‐Meier curves. However, only one to two patients with plasma levels above the cut‐off for the protein in question died due to CRC, while multiple patients with levels below the cut‐off died due to CRC, leading to slightly unbalanced groups. FETUB did not have a significant p‐value when analyzed by Cox regression analysis when stage II samples were compared (*P* = .053), although the Kaplan‐Meier curve drawn (Figure 2E) showed significant differences in outcome as analyzed by log‐rank test (*P* = .003).

### 3.4 Analysis of stage III samples

When samples from only patients with stage III CRC were analyzed by Cox regression, five proteins had significantly different *P*‐values (Table S4). Two of the proteins, MORC family CW‐type zinc finger protein 2 (MORC2) and phosphatidylcholine‐sterol acyltransferase (LCAT) had HRs of <1, indicating that high plasma levels of these two proteins predicted a favorable outcome. However, the HR for the other three proteins, mannose‐binding protein C (MBL2), signal‐induced proliferation‐associated 1‐like protein 1 (SIPA1L1), and phosphatidylinositol‐glycan‐specific phospholipase D (GPLD1), were higher than 1, indicating that high plasma levels are a marker of poor survival for stage III CRC patients. These samples were also adjusted for age and analyzed by Cox regression, after which three proteins with significant *P*‐values were found (Table S5). Two of these proteins, LCAT and SIPA1L1, also had significant *P*‐values prior to age‐adjustment, indicating that they could be of value in predicting outcome. The third protein, ephrin type‐A receptor 5 (EPHA5), had a non‐significant *P*‐value prior to age‐adjustment, although higher levels were subsequently linked to a favorable outcome in stage III patients afterwards.

**Figure 3:**
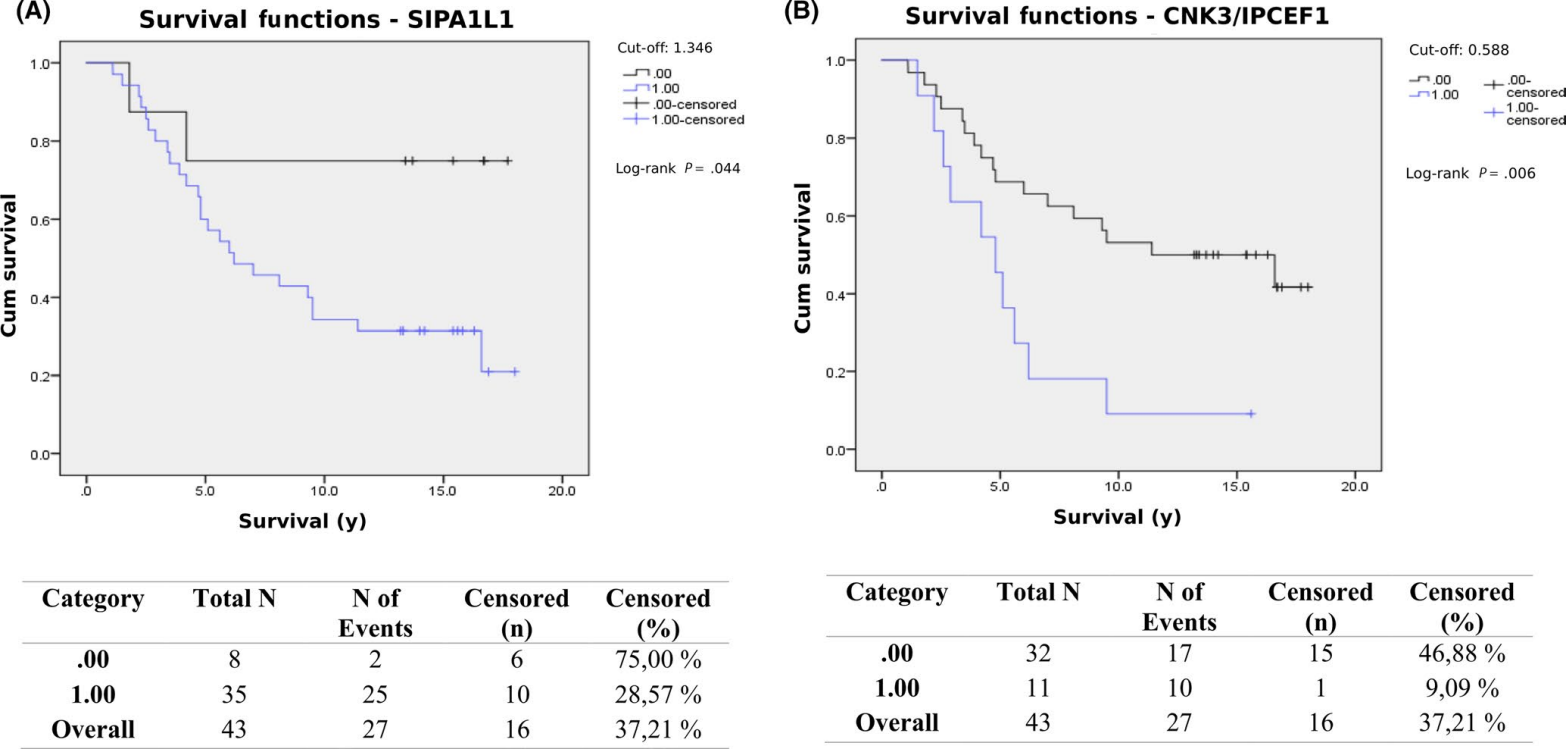
The Kaplan‐Meier curves for SIPA1L1 (A) and CNK3/IPCEF1 (B) when only stage III samples were analyzed. The cut‐off was set at the maximum of Youden's index for each protein. Plasma levels above the cut‐off for both proteins are correlated with poor outcome in stage III CRC patients

Again, Kaplan‐Meier curves were drawn with the cut‐off set at the maximum of Youden's index for each protein. The Kaplan‐Meier curve for SIPA1L1, shown in Figure 3A, confirms the findings by Cox regression analysis that higher plasma levels of SIPA1L1 are correlated with poor outcome in stage III CRC patients. Only two patients with plasma levels of SIPA1L1 below the cut‐off died due to CRC, compared to 25 patients with levels that were above the cut‐off that died due to CRC. Although not significant when analyzed by Cox regression analysis (*P* = .08), Kaplan‐Meier analysis showed that for stage III patients, higher plasma levels of the CNK3/IPCEF1 fusion protein (CNK3/IPCEF1) lead to a significantly (*P* = .006 as analyzed by log‐rank test) poorer long‐term survival rate, especially as time progressed (Figure 3B).

## 4 DISCUSSION

In this study, we identified several plasma proteins that could aid in predicting the outcome of CRC patients. When all samples were analyzed by univariate Cox regression, higher levels of plasma proteins such as CP, TVP23C, FETUB, and IGFBP3 (*P* < .05) were each found to predict a favorable outcome. When they were adjusted for both age and stage and re‐analyzed, FETUB was the only protein with a significant *P*‐value. Additionally, Kaplan‐Meier curves further supported these findings, showing that patients with high levels of CP, TVP23C, or IGFBP3 had a significantly higher long‐term survival rate (Figure 1). The difference in survival was not significant for patients with high or low levels of FETUB when analyzed by Kaplan‐Meier. High levels of TVP23C were also found to correlate with a favorable outcome when stage II samples were analyzed separately, both before and after being adjusted for age. When stage III samples were analyzed separately by Cox regression, LCAT and SIPA1L1 had significant p‐values both before and after age‐adjustment. High plasma levels of LCAT were correlated with a favorable outcome, while high levels of SIPA1L1 were correlated with a poor outcome for stage III CRC patients. LCAT and FETUB are proteins involved in metabolism, with roles in lipid and glucose metabolism respectively. Metabolism is altered in cancer cells, enabling their survival and proliferation, and the reprogramming of metabolism by cancer cells has been recognized as a hallmark of cancer.^27^, ^28^

In concordance with our findings that higher levels of IGFBP3 were linked to a favorable outcome in this set of CRC patients, a previous study found that men with higher plasma levels of IGFBP3 had a lower risk of developing CRC.^29^ IGFBP3 binds insulin‐like growth factors (IGFs) and subsequently influences proliferation by modeling the access of IGFs to their receptors, and has also been shown to induce apoptosis in a prostate cancer cell line through an IGF‐independent mechanism.^29^, ^30^ Serum levels of C3 have previously been found to be higher in CRC patients compared to healthy controls,^31^ implying that measuring levels of C3 may aid in the diagnosis of CRC, although there are no previous studies investigating the value of C3 as a prognostic marker. Here, we found that higher plasma levels of C3 were linked to a favorable outcome, although only in stage II patients. CNK3/IPCEF1 is a fusion protein that is required for hepatocyte growth factor (HGF)‐mediated Arf6 activation and HGF‐dependent migration.^32^ Arf6 is a protein involved in cell adhesion and migration, and Arf6 activation is required for the motile phenotype of epithelial cells. Arf6 activation has been shown to be important for cancer cell proliferation, invasion, and metastasis in various cancers, with inhibition of Arf6 suppressing invasion and metastasis.^33^, ^34^ This may help explain our findings that plasma levels of CNK3/IPCEF1 are higher in stage III CRC patients with poor outcome. To the best of our knowledge, it has not been previously shown that altered levels of proteins such as FETUB, TVP23C, LCAT, SIPA1L1, and CNK3/IPCEF1 are correlated with good or poor outcome in CRC patients.

This study was strengthened by the fairly large number of samples analyzed and the rigorous inclusion criteria used, where patients with other types of cancer and underlying diseases that likely affect plasma protein expression, such as inflammatory bowel disease, were excluded. This study was also strengthened by the well‐characterized patient cohort and the long follow‐up times for the patients included, as some CRC patients relapsed and eventually succumbed to their disease up to 9 years after their initial operation, and other patients were disease‐free and were followed for up to 18 years after their operation. One limit of this study was the lack of validation of the proteins identified in an independent cohort of patients. Further validation and additional studies are necessary to investigate if these plasma proteins can be of use in the clinic and why high or low levels are linked to longer or shorter survival times. Additionally, when samples were divided according to stage, the number of patients in each group was reduced, which should also be taken into account.

One advantage of the proteins identified in this study is that because they are plasma proteins, their levels can non‐invasively be measured. However, the largest differences in long‐term survival were seen after 10+ years of follow‐up, indicating that while these proteins may be of use for monitoring patient status, they may not be ideal for predicting relapse at an early stage. Additionally, as the proteins identified are plasma proteins and not tumor‐specific proteins, their levels can also be affected by other conditions and diseases besides CRC, which must be taken into account. Here, we identified multiple plasma proteins such as CP, TVP23C, FETUB, and IGFBP3 that, after further studies and validation, could be measured during patient follow‐up and monitoring to assist in predicting outcome.

## CONFLICT OF INTEREST

The authors declare no conflict of interest.

## AUTHOR CONTRIBUTIONS

MH, SJ, MS, AR, RR, and CH conceived and designed the study. MH and CH collected the plasma samples as well as the patients’ clinical and survival data. MH, SJ, and TT acquired the mass spectrometric data. MH, SJ, MS, and HM analyzed and interpreted the data. MH wrote the manuscript. All authors revised the manuscript. AR, RR, and CH provided resources.
