# Supplementary material for: Identification of several plasma proteins whose levels in colorectal cancer patients differ depending on outcome
Source: FASEB Bioadv. 2019 Nov 23;1(12):723–30. doi: 10.1096/fba.2019-00062 (PMC6996405; doi:10.1096/fba.2019-00062)
Supplement: Supplementary file 1 [file FBA2-1-723-s001.pdf]

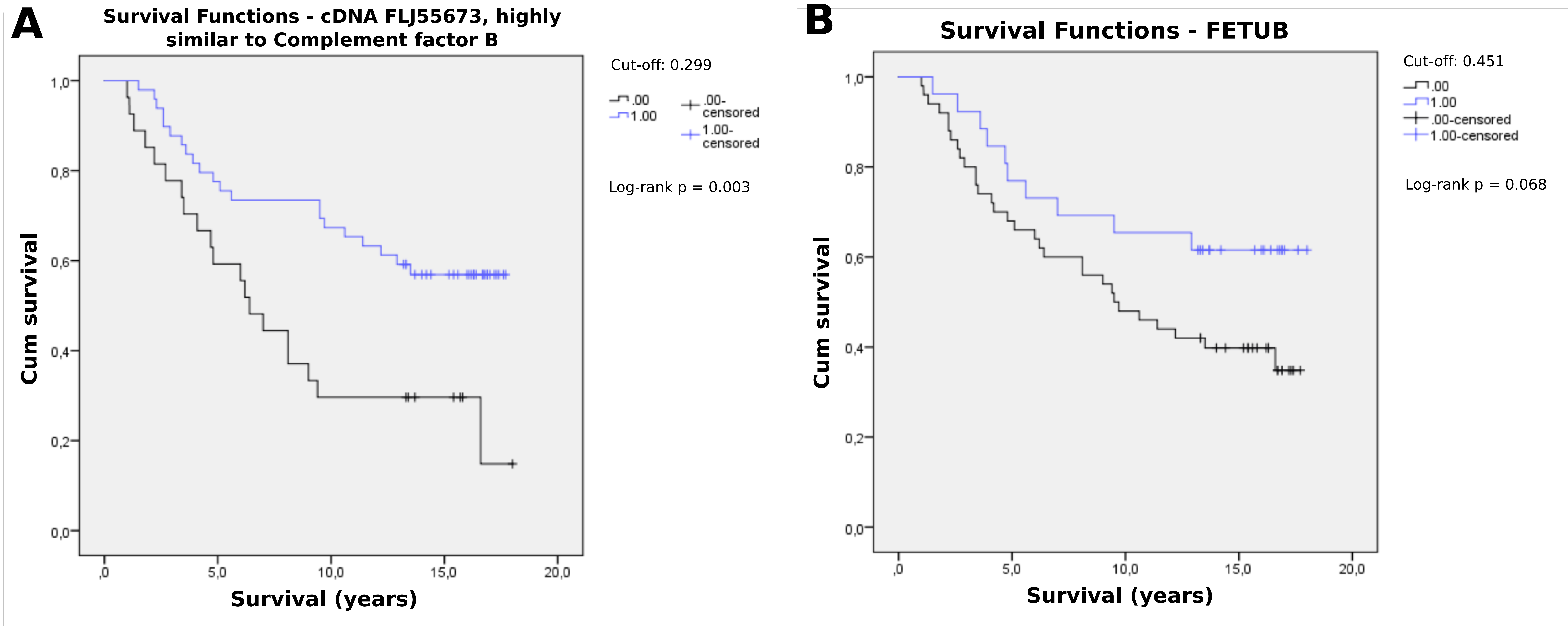

Category	Total N	N of Events	Censored (n)	Censored (%)
**.00**	27	20	7	25,93 %
**1.00**	49	21	28	57,14 %
**Overall**	76	41	35	46,05 %

Category	Total N	N of Events	Censored (n)	Censored (%)
**.00**	27	20	7	25,93 %
**1.00**	49	21	28	57,14 %
**Overall**	76	41	35	46,05 %
